# Use of a Blood Biomarker Test Improves Economic Utility in the Evaluation of Older Patients Presenting with Cognitive Impairment

**DOI:** 10.1089/pop.2023.0309

**Published:** 2024-06-15

**Authors:** William J. Canestaro, Randall J. Bateman, David M. Holtzman, Mark Monane, Joel B. Braunstein

**Affiliations:** ^1^ Department of Management and Organization, Foster School of Business, University of Washington, Seattle, Washington, USA.; ^2^ Department of Neurology, Washington University School of Medicine in St. Louis, St. Louis, Missouri, USA.; ^3^ C_2_N Diagnostics, LLC, St. Louis, Missouri, USA.

**Keywords:** Alzheimer's disease, blood biomarker, diagnosis, economic utility, budget impact model, cost analysis

## Abstract

More than 16 million Americans living with cognitive impairment warrant a diagnostic evaluation to determine the cause of this disorder. The recent availability of disease-modifying therapies for Alzheimer's disease (AD) is expected to significantly drive demand for such diagnostic testing. Accurate, accessible, and affordable methods are needed. Blood biomarkers (BBMs) offer advantages over usual care amyloid positron emission tomography (PET) and cerebrospinal fluid (CSF) biomarkers in these regards. This study used a budget impact model to assess the economic utility of the PrecivityAD^®^ blood test, a clinically validated BBM test for the evaluation of brain amyloid, a pathological hallmark of AD. The model compared 2 scenarios: (1) baseline testing involving usual care practice, and (2) early use of a BBM test before usual care CSF and PET biomarker use. At a modest 40% adoption rate, the BBM test scenario had comparable sensitivity and specificity to the usual care scenario and showed net savings in the diagnostic work-up of $3.57 million or $0.30 per member per month in a 1 million member population, translating to over $1B when extrapolated to the US population as a whole and representing a 11.4% cost reduction. Savings were driven by reductions in the frequency and need for CSF and PET testing. Additionally, BBM testing was associated with a cost savings of $643 per AD case identified. Use of the PrecivityAD blood test in the clinical care pathway may prevent unnecessary testing, provide cost savings, and reduce the burden on both patients and health plans.

## Introduction

More than 16 million Americans are living with cognitive impairment and warrant a diagnostic evaluation to determine the cause of their signs and symptoms of cognitive decline.^1^ Among affected patients, the classifications include mild cognitive impairment (MCI, ∼9 MM) and dementia (∼7 MM) individuals. Alzheimer's disease (AD) is the underlying cause in 30% and 60% of cases, respectively.^2^ The economic toll for this disease within the United States amounts to more than $345 billion in 2023.^3^

The standard of care for clinical evaluation of patients presenting with cognitive impairment includes a comprehensive patient history, physical exam, and neurocognitive testing (NCT). Yet, these clinical evaluation methods have fundamental limitations. Although noninvasive in nature, the history and physical exam are of limited usefulness in aiding the health care provider to rule out or rule in AD as the cause of the underlying symptoms.^4^ If abnormalities are found, the next step is a referral to the neurologist/memory specialist as well as obtain brain imaging such as computed tomography or magnetic resonance imaging scans. These scans look for structural changes such as tumors and strokes; however, they do not specifically confirm an AD diagnosis.^5,6^ The addition of other biomarker tests, including amyloid positron emission tomography (PET) imaging and/or lumbar puncture with cerebrospinal fluid (CSF) analysis, have greatly increased the diagnostic accuracy of the evaluation for AD.

With regards to patient treatment, symptomatic pharmacotherapy with only modest clinical benefit was available until recently. Both acetylcholinesterase inhibitors as first-line therapy for mild-to-moderate disease and the N-methyl-d-aspartate receptor agonist memantine for moderate-to-severe symptoms^7^ relieve symptoms without clearly altering disease progression. However, recently published results from the phase 3 trials for lecanemab^8^ and donanemab^9^ have shown that removing amyloid deposits from the brain decelerates the progression of AD when used in early disease stage such as MCI and mild dementia. Subsequently, lecanemab was approved as the first drug to target the underlying biology of disease in the United States and Japan.

The approval of these new disease-modifying treatment (DMT) options creates significant challenges for health care systems because of the unique combination of a large and largely undiagnosed prevalence pool of patients as well as a complex diagnostic process to determine treatment eligibility. A formal determination of AD as the underlying etiology of the patient's symptoms requires identification of the presence of brain amyloid pathology with amyloid PET imaging or CSF analysis. Neither diagnostic modality can be easily scaled to meet the expected demand.^10^ Most clinics operate their PET scanners at near-capacity, mostly for oncology patients: installing new devices requires years of planning and sizeable capital investment.^11^ Further, the instability of the radioactive amyloid tracer limits its use to the area reasonably close to a cyclotron, which deprives less populated areas of access to amyloid PET imaging, even if mobile PET devices allowed for conducting scans. While less constrained by geographic access, lumbar punctures are not widely embraced in the United States because of their invasive nature.

As more patients seek a diagnosis to determine their eligibility for treatment, the combination of limited capacity for AD biomarker testing and sole reliance on brief cognitive testing is expected to lead to prolonged specialist wait times. Delays of up to 50 months are projected, which in turn causes avoidable disease progression, potentially to a stage at which DMTs are no longer effective.^12^ Blood biomarker (BBM) tests for the evaluation of AD pathology have emerged as potential solution, as such options are considerably more scalable and accessible than PET imaging and CSF analysis.^13^

While the analytical validity,^14^ clinical validity,^15^ and clinical utility^16^ of the PrecivityAD^®^ (C_2_N Diagnostics, LLC, St Louis, MO, USA) blood test, commercially available since 2020, have been demonstrated in multiple studies versus amyloid PET as the reference standard, the economic utility and budget impact of the PrecivityAD test have not been well described in the literature.

The objective of this study, designed to complement the earlier work described above, is the evaluation and calculation of the economic utility of the implementation of a novel diagnostic test for Alzheimer's pathology, the PrecivityAD blood test, into a clinical care pathway. The economic model compared 2 scenarios to aid in the assessment of the presence or absence of brain amyloid plaques, a pathological hallmark of AD: (1) baseline testing involving usual care practice of CSF biomarker and PET biomarker tests, and (2) early use of a BBM test, followed by CSF biomarker and PET biomarker testing dependent on the BBM test result.

## Materials and Methods

### BBM test output and performance

The PrecivityAD blood test is intended for use in patients aged 55 and older with signs or symptoms of MCI or dementia undergoing evaluation for Alzheimer's disease or other causes of cognitive decline. The test quantifies an individual's plasma Aβ42/40 Ratio and identifies apolipoprotein E isoform-specific peptides (ApoE Proteotype) to infer *APOE* genotype using high resolution tandem liquid chromatography mass spectrometry.^14^ A proprietary algorithm combines Aβ42/40 ratio, ApoE Proteotype, and age to derive the test readout, Amyloid Probability Score (APS) from 0 to 100: the higher the APS result, the more likely the patient is to have a positive amyloid PET scan.

The clinical validity of this BBM test was evaluated among 686 patients with MCI or dementia and an amyloid disease prevalence rate of 55.1% from the Plasma Test for Amyloidosis Risk Screening study (a subset of the IDEAS Study, ClinicalTrials.gov Identifier: NCT02420756) and the MissionAD study (derived from the elenbecestat Phase 3 trials, ClinicalTrials.gov Identifier: NCT02956486) cohorts.^15^ As a rule-out test, the PrecivityAD blood test showed a 92% sensitivity and 86% negative predictive value for Low APS (0–35). As a rule-in test, the PrecivityAD blood test showed a 77% specificity and 86% positive predictive value for High APS (58–100). The Area under the Curve for the Receiver Operating Characteristic was 0.88, and the overall accuracy relative to amyloid PET status was 85%. These values excluded the ∼14% of patients who fell into the Intermediate score (36–57) category, where the test cannot distinguish between the presence or absence of brain amyloid plaques.

The Quality Improvement and Clinical Utility PrecivityAD Clinician Survey (QUIP I) study (ClinicalTrials.gov Identifier: NCT05477056) demonstrated the clinical utility of this BBM test.^16^ The final analysis included 366 patients (median age 75 years, 56% women) across 15 different US clinic sites and 43 different health care providers. A 95% (347/366) concordance rate was noted between clinicians' patient selection and the test's intended use criteria. Prespecified test result categories included 38% Low APS (*n* = 133), 47% High APS (*n* = 162), and 15% Intermediate APS (*n* = 52) patients. Clinicians' pre- and posttest AD diagnosis probability changed from 58% to 23% for Low APS patients and 71% to 89% in High APS patients (*P* < 0.0001). Overall, AD drug therapy was changed for 33% of patients from pre- to posttesting: drug therapy was reported reduced in 46% of Low APS patients (*P* < 0.0001) and increased in 57% of High APS patients (*P* < 0.0001).

### Model structure and target population

**FIG. 1.**
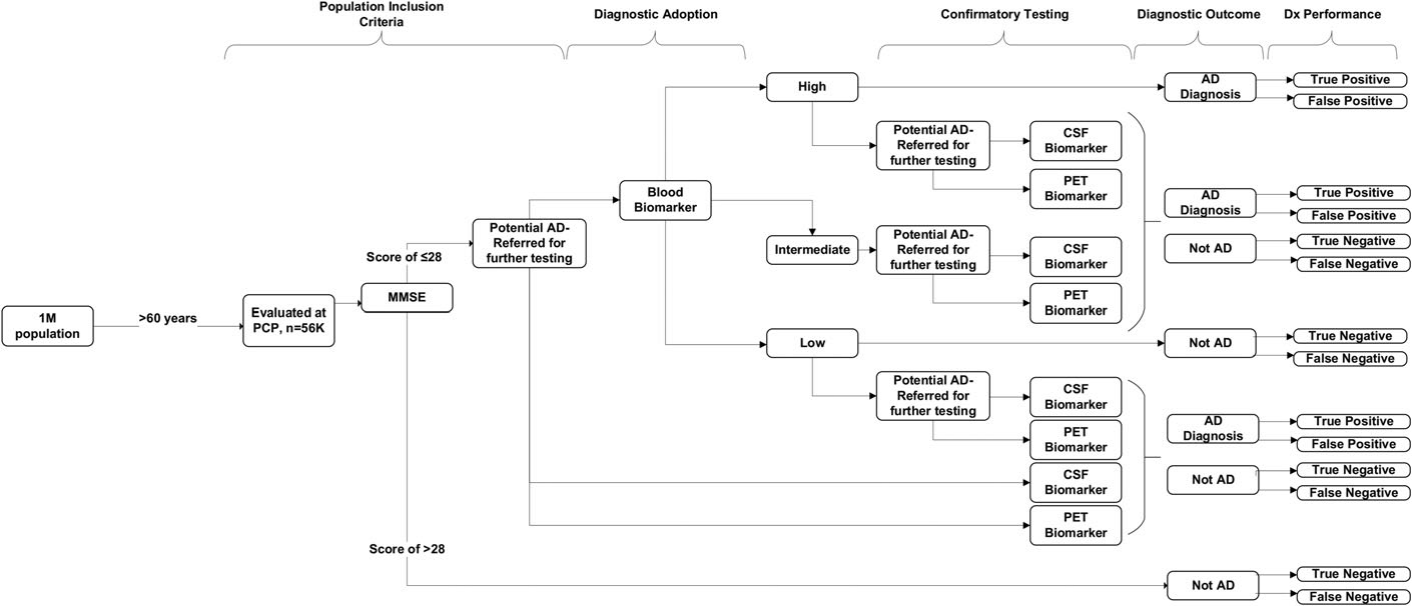
Model structure. The 2 diagnostic scenarios are illustrated. The model compared 2 scenarios: (1) baseline testing involving usual care practice, and (2) early use of a BBM test with referral to PET biomarker and CSF biomarker use depending on the BBM result. Patients presenting to the primary care setting and memory specialist setting with abnormal MMSE progress down each diagnostic pathway. High, Intermediate, and Low refer to blood biomarker test results. AD, Alzheimer's disease; BBM, blood biomarker; CSF, cerebrospinal fluid analysis; Dx, diagnostic; MMSE, Mini-Mental State Examination; PCP, primary care provider; PET, positron emission tomography.

The model structure was a decision-tree that considered a hypothetical 1 million member US health plan 1 year after preferentially introducing the BBM testing in the clinical care pathway for the evaluation of patients aged 60 and older presenting with cognitive impairment (Fig. 1). Although the PrecivityAD test has been clinically validated in populations aged 55 years and older, the authors focused the analysis here on populations 60 years and older to enrich the target population with MCI and dementia, rather than subjective cognitive dysfunction that is more prevalent among 55–60 year olds.^17^ The entry point into the model was an abnormal cognitive assessment among patients evaluated in the primary care setting. This decision-tree model was focused on a budget impact analysis that evaluated outcomes over a 1-year period and did not consider outcomes beyond 1 year. Real-world adoption and adherence values were included and extrapolated from peer-reviewed, clinical literature.

The model compared 2 scenarios: baseline usual care testing and BBM testing. Patients with signs or symptoms of cognitive impairment were evaluated using a NCT for the evaluation of cognitive impairment. Those patients with abnormal findings were then advanced to either of the 2 following scenarios. The baseline usual care scenario was modeled after current practice, and patients were evaluated with either a CSF or PET biomarker test. This baseline usual case test scenario assumed no adoption of the BBM test. The alternative, BBM test scenario allowed for a modest level of PrecivityAD test adoption in the primary care setting as a first evaluation. The majority of BBM adoption in this scenario was modeled to occur at the neurologist/memory care specialist's office after a referral. Furthermore, the BBM test scenario included follow-up CSF or PET biomarker testing depending on the BBM test result (Table 1). The model followed patients to their diagnostic outcome only and did not consider lifetime costs or outcomes of patients based on their diagnostic result.

**Table 1.** Clinical Inputs into the Model

Variable	Value (%)	References
Percent of population over 60 years of age	22	^30^
AD screening at PCP by MMSE	25	^31^
BBM adoption by PCP	5	Assumption
Referred to neurologist/memory specialist after BBM testing by PCP		
High score	100	Assumption
Intermediate score	100	Assumption
Low score	20	Assumption
Referred to secondary testing despite negative MMSE/NCT	20	Assumption
Referred to secondary testing after BBM testing by neurologist/memory specialist		
High score	20	Data on file
Intermediate score	100	Assumption
Low score	20	Data on file
Secondary test use		
CSF analysis	20	Assumption
PET imaging	80	Assumption
Diagnostic uptake		
BBM testing	40	Assumption
CSF analysis	12	Assumption
PET imaging	48	Assumption
Dropout		
After PCP	20	Assumption
After NCT	20	Assumption
MMSE		
Sensitivity	82	^32,33^
Specificity	73	^32,33^
Test–retest reliability	87.5	^32,33^
Result prevalence		
>28	76	Calculation
21–28	15	^22,34^
<21	9	^22,34^
CSF analysis test performance		
Sensitivity	81	^35^
Specificity	90	^35^
Result prevalence		
Positive	61	^36^
Negative	39	^36^
Confirmatory result prevalence		
Positive	90	Assumption
Negative	10	Assumption
PET imaging performance		
Sensitivity	92	^37^
Specificity	95	^37^
Result prevalence		
Positive	62	^38^
Negative	38	^38^
Confirmatory result prevalence		
Positive	90	^39^
Negative	10	^39^
BBM test performance		
Sensitivity	92	^15^
Specificity	77	^15^
Result prevalence		
High	47	^16^
Intermediate	15	^16^
Low	38	^16^

AD, Alzheimer's disease; BBM, blood biomarker; CSF, cerebrospinal fluid analysis; MMSE, Mini-Mental State Examination; NCT, neurocognitive testing; PCP, primary care provider; PET, positron emission tomography.

### Clinical inputs

Clinical inputs into the model were drawn from the clinical literature as well as assumptions based on this literature when necessary (Table 1). Demographic characteristics were drawn from the US Census findings. Behavioral inputs such as the percent of patients referred and lost to follow-up were elicited from assumptions based on patterns of care in the AD clinical evaluation space. The model included realistic rather than idealized characteristics to more accurately reflect test utilization in the outpatient care setting.

In the primary care provider (PCP) setting, it was assumed that only 25% of eligible patients would be evaluated for cognitive decline via the Mini-Mental State Examination (MMSE). Following this initial evaluation, the model assumed that 5% of patients with scores in the 21–28 range for MMSE would be tested with a BBM in the primary care setting (Fig. 1). Patients with MMSE scores of 28 and below are referred to a neurologist/memory specialist for further clinical evaluation. Once at the neurologist/memory specialist, patients receive a NCT assessment to confirm results from the PCP. The model assumed 20% drop-out from the PCP to NCT due to loss to follow-up as well as 20% dropout due to loss to follow-up from neurologist to CSF biomarker or PET biomarker testing. Several of the study inputs were derived from the QUIP I study of the clinical utility of the PrecivityAD blood test.^16^

The effects of increasing adoption of the BBM test at the neurologist office were studied using a 40% adoption rate as the main focus and default for the budget impact modeling. A sensitivity analysis assessed this assumption by evaluating test adoption from 0% to 100%.

High and Low APS test results on the BBM tests were projected to be evaluated by follow up testing in 20% and 20% of cases, respectively. Intermediate APS test results were projected to go on to CSF biomarker or PET biomarker analysis in 100% of cases. Patients who did not receive the BBM test were assumed to have an 80% PET testing/20% CSF testing distribution as the method to determine the presence or absence of brain amyloid.

### Economic inputs

Economic inputs into the model were described as costs in 2023 US dollars and, when appropriate, adjusted for inflation via the health care inflation rate from the Consumer Price Index (Table 2). The study team calculated the costs incurred from Medicare payer perspective for each diagnostic strategy. The model included costs for cognitive assessment (MMSE and other neuropsychological testing) as well as procedure costs including CSF biomarker testing (including costs of lumbar puncture, neurologist/memory specialist visits, and CSF analysis), PET biomarker testing (including costs of the amyloid PET tracer, technical components of the amyloid PET scan, and professional component of the amyloid PET scan), and BBM testing (including costs of venipuncture and PrecivityAD testing). Because these costs are known to vary by payer and geographic region, the model evaluated the impact of varying costs in sensitivity analyses.

**Table 2.** Economic Inputs Into the Model

Variable	Cost	Reference^40^
Provider cost		
MMSE and neurocognitive testing	$149	CPT 96125
Physician office visit	$233	CPT 99205
Procedure cost		
Venipuncture	$17	CPT 36410
Lumbar puncture	$390	CPT 62270 blended rate
CSF assay	$311	PLA 0385U, CPT 82542
Amyloid PET—technical component	$1,346	CPT 78811
Amyloid PET—professional component	$70	CPT 78811
Amyloid PET—diagnostic radiopharmaceutical	$2,964	HCPCS A9586, Q9982, Q9983
BBM test	$1,250	Data on file

BBM, blood biomarker test; CSF, cerebrospinal fluid analysis; MMSE, Mini-Mental State Examination; PET, positron emission tomography.

### Study outcomes

The study outcomes included diagnostic outcomes as well as cost outcomes. For the diagnostic outcomes, the model tracked the measurement characteristics of each diagnostic pathway and compared overall sensitivity and specificity for the usual care and BBM testing scenarios. The number of patients diagnosed were also compared between the 2 scenarios.

For cost outcomes, costs for diagnostic testing and clinic visits were measured. The key cost impact outcome of this model was the overall cost for care between the baseline and BBM test scenario. Another key cost outcome variable was the incremental difference in cost per case identified between the baseline and the PrecivityAD blood test scenario. This outcome is defined below:

$$\begin{array}{cc} & Incrementalcostpercaseidentified\\ & =\frac{\begin{array}{cc} & TotalCosts_{PrecivityADScenario}\\ & -TotalCosts_{ControlScenario} \end{array}}{\begin{array}{cc} & CasesIdentified_{PrecivityADScenario}\\ & -CasesIdentified_{ControlScenario} \end{array}} \end{array}$$

This incremental cost per case identified is reported as a negative value in such scenarios where the BBM testing is less expensive than the comparator testing and is reported as a positive value where the BBM testing is more expensive than the comparator testing. Per member per month (PMPM) costs and savings associated with 2 scenarios were also calculated.

### External validity and sensitivity analyses

The economic model was checked with extreme values testing and comparison to previously published models to ensure that the model could replicate previous results for other AD biomarkers as closely as possible.^18–20^ In addition, the model was designed to allow for probabilistic sensitivity analysis.

For these sensitivity analyses, each input parameter was assigned a distribution and then modified across a range of values to estimate the impact of the uncertainty in 1000 simulations of the model. With this process, normal distributions were used for clinical counts, and average cost data and beta distributions were used for probabilities. Probabilistic cost and count input parameters were bounded at 0 to ensure positive values. All inputs were varied across a ±10% range unless a confidence interval was available from the original literature. The values were reported in overall costs for each scenario and the overall measurement characteristics for the whole scenario. This probabilistic savings analyses provide for a population estimate of effect size, and incremental costs between the 2 scenarios were also considered: *P*-values were derived using *t*-test statistics for comparison of means.

## Results

### Diagnostic outcomes

Based on a million member health plan, the model included 56,558 members aged 60 or older who received MMSE testing for evaluation of cognitive impairment. The model showed that a clinical pathway using the BBM test at a 40% adoption rate of the BBM test had specificity comparable with current standard of care (99.27% vs. 98.75%). Sensitivity was also similar in the 2 scenarios, with sensitivity of 34.87% for the BBM scenario and sensitivity of 35.31% for the baseline CSF biomarker or PET biomarker scenario. The number of patients diagnosed with AD in the 2 scenarios were comparable as well, with 4814 and 4910 patients, respectively, in the usual care scenario versus BBM scenario.

### Budget impact model and cost per case identified

The BBM scenario was associated with cost savings, primarily driven by reductions in CSF biomarker and PET biomarker testing (Table 3). Under the usual care scenario, the cost to the health plan was $31.3 million. Incorporating the BBM test in the scenario with a 40% adoption rate was associated with a cost of $27.7 million, resulting in cost savings of $3.57 million (11.4% reduction in costs). Costs for overall CSF biomarker and PET biomarker testing fell by 28%, leading to $437,000 savings and $7.4 million savings, respectively, under the BBM scenario, which included the use of CSF biomarkers and PET biomarkers either as primary tests before BBM testing or as secondary tests after BBM testing. These reductions in CSF biomarker and PET biomarker testing costs were greater than the $4.4 million cost generated by the addition of the BBM testing itself. In addition, the cost per case identified decreased by 9.3% ($643/$6,948) at a 40% adoption rate for the BBM test. The breakeven price for a BBM test with the test performance and clinical inputs described in this analysis was $2,272.

**Table 3.** Budget Impact Model Results for the Diagnostic and Cost Outcomes

	Baseline	BBM @ 40% adoption	
		Results	Incremental
Diagnostic outcomes			
Patients diagnosed	4792	4888	96
Sensitivity (%)	35.31	34.87	−0.43
Specificity (%)	99.27	98.75	−0.51
Cost outcomes			
Clinic visits	$ 2,886,844	$ 2,874,445	$ 12,399
BBM testing	$ −	$ 4,367,139	$ (4,367,139)
CSF biomarker testing	$ 1,567,993	$ 1,130,638	$ 437,355
PET biomarker testing	$ 26,849,741	$ 19,360,636	$ 7,489,106
Total	$ 31,304,578	$ 27,732,857	$ 3,571,721
Cost per case identified	$ 6,948	$ 6,305	$ 643

BBM, blood biomarker test; CSF, cerebrospinal fluid analysis; PET, positron emission tomography.

**FIG. 2.**
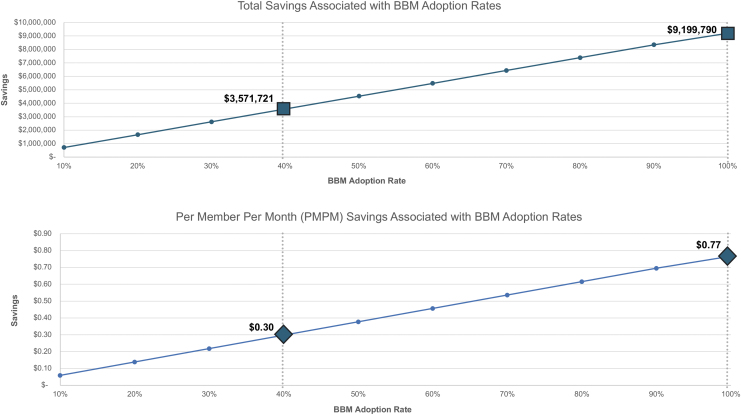
Total savings and per member per month savings associated with BBM adoption rates. Overall, cost savings and PMPM savings derived from the implementation of the blood biomarker care scenario care are plotted against blood biomarker adoption rates. BBM, blood biomarker; PMPM, per member per month.

The adoption of the BBM test at 40% of eligible patients receiving BBM testing for AD pathology represented the base case for the BBM test scenario. At this level of adoption, overall cost savings to the plan was $3.57 million or $0.30 PMPM (Fig. 2). Health plan savings and PMPM savings increased proportionally to the adoption of the BBM testing. At a hypothetical 100% adoption rate for the BBM test in the evaluation of patients presenting with cognitive impairment, the BBM scenario was associated with increased savings over the baseline usual care scenario to $9.2 MM or $0.77 PMPM.

### Probabilistic sensitivity analyses

**FIG. 3.**
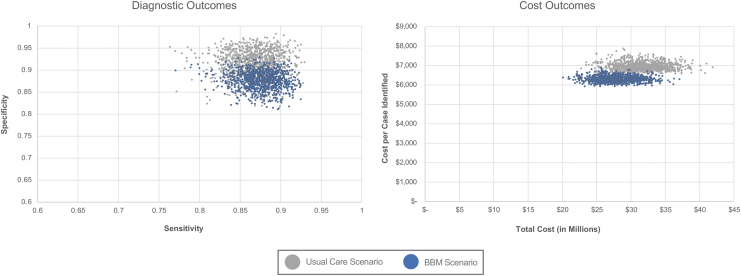
Probabilistic sensitivity analysis of diagnostic and cost outcomes. For the diagnostic outcomes analysis, specificity is plotted against sensitivity for the usual care versus blood biomarker care scenarios based on 1000 simulations of the model. For the cost outcomes analysis, cost per AD case identified is plotted against the total cost of care for the usual care versus blood biomarker care scenarios based on 1000 simulations of the model. AD, Alzheimer's disease; BBM, blood biomarker.

For these sensitivity analyses, each input parameter was assigned a distribution and then modified across a range of values to estimate the impact of the uncertainty in 1000 simulations of the model (Fig. 3). With regards to the sensitivity and specificity of the overall diagnostic care pathway, the results for the probabilistic sensitivity analysis showed significant overlap in diagnostic outcomes between the BBM test and baseline scenario, suggesting that there were numerous scenarios where the BBM scenario outperformed the usual care scenario. The confidence interval for the differences in both sensitivity and specificity failed to exclude the null.

With regards to the costs of care of the overall diagnostic pathway, costs were much more discretely separated from the initial distribution values, suggesting greater certainty in the robustness of the cost saving model of the BBM test. All cost savings described above were statistically significant at the *P* < 0.0001 level in this probabilistic sensitivity analysis.

## Discussion

This economic model analysis suggests a substantial cost savings and favorable budget impact for the BBM test scenario compared to usual care CSF biomarker and PET biomarker test scenario to a hypothetical health plan in a budget impact analysis using Medicare cost inputs. The savings were robust across several variables, including cost of the overall evaluation of patients presenting with cognitive impairment (11.4% lower costs), PMPM cost of testing ($0.30 PMPM savings across the 1 million member health plan), and cost per case identified in this budget impact model study (9.3% savings).

These savings were largely driven by savings in CSF biomarker and PET biomarker testing costs, which were deferred or ruled-out based on the BBM test results. The potential for economic impact is significant: when extrapolated to the entire US population of 330 million individuals, the implementation of the PrecivityAD blood test, the BBM test used depicted in this model, in the evaluation of patients presenting with cognitive impairment or dementia undergoing evaluation for AD or other cause of cognitive decline would result in greater than $1B ($3.57 million in savings in a 1 MM member population × 330) in savings at a 40% adoption rate based on the modeling.

Importantly, these cost savings outlined above come without any compromise in quality of care, since the adoption of the BBM test achieved comparable diagnostic outcomes as that seen in the baseline scenario with CSF biomarker and PET biomarker testing. Thus, the PMPM savings in this study are noteworthy, as implementation strategies for novel technologies most often require tradeoffs such as a gain in clinical benefit although at a higher cost of care.^21^ In this study, instead of a PMPM increase, the BBM testing scenario enabled PMPM savings. Furthermore, the breakeven price for biomarker testing in this analysis ($2,272) was 81% higher than that of the current price of BBM test cost that the authors used as model ($1,267), suggesting that the current pricing provides strong value to the health care system. This economic utility of the PrecivityAD blood test presented in this work represents a complement to the evidence of clinical utility of the test and its association with changes in clinician decision-making associated with the PrecivityAD blood test results.

There are several current and emerging trends that necessitate the need for improved strategies for the evaluation of cognitive impairment. A longer lifespan has led to a significant rise in the number of patients with cognitive impairment: a recent review found the prevalence of MCI and dementia to be ∼24% among individuals 65 years and older in the United States.^22^ Driven by population aging, the number of Americans with dementia is expected to increase significantly in the next 3 decades, placing major cost demands for diagnostic and treatment services on the health care system. There is increasing evidence on the value of early diagnosis of AD.^23^ The incentive to seek a formal diagnosis and begin treatment early in the course of disease progression will likely increase given the recent availability of new DMTs for AD,^24^ as the full Food and Drug Administration (FDA) approval and coverage decision by the Centers for Medicare and Medicaid Services in July 2023 of the new anti-amyloid drug lecanemab (Leqembi^®^) mandates the amyloid pathology confirmation of disease before initiating drug therapy.

The cost impact of biomarker testing has been described for neurological diseases^18–20,25^ as well as other chronic diseases,^26,27^ showing the economic utility of such approaches. The budget impact model findings for a BBM in the evaluation of cognitive impairment related to AD or other causes of cognitive decline as reported in this study provide the environment for health care providers and health plans to address a major unmet need for safe, lower cost, less resource-intensive, easily accessible, and broadly available test that can identify the presence or absence of brain amyloid plaques, a pathological hallmark of AD as an alternative method to usual care CSF and PET biomarkers for assessing brain amyloid.^13^ Furthermore, the International Society for Pharmacoeconomics and Outcomes Research 2012 Budget Impact Analysis Good Practice II Task Force recommended that budget impact model calculations should be performed by using a simple cost calculator approach because of its ease of use for budget holders.^28^

The analysis used in this study focused on a series of data inputs that can be entered and modified accordingly to align with the characteristics of a given health plan. Since clinical inputs for the BBM used in this study were derived from clinical validity and clinical utility studies conducted with the PrecivityAD blood test, a budget impact model based on other BBMs would benefit from real-world data on biomarker use and clinical decision-making after BBM testing as used in this analysis.

There are several limitations to this analysis. First, this analysis made numerous assumptions around inputs into the budget impact analysis. For example, the clinical pathway for patients following the results of Low, High, and Intermediate APS test results from BBM testing may vary from the published literature. While the current modeling scenarios based on 1 year follow-up and 1 time evaluation of patient in the diagnostic pathway are grounded in real-world utilization data derived from the clinical literature, the modeled changes in utilization of the CSF and PET biomarker testing are hypothetical, yet meant to realistically explore what would happen if a health plan were to change the combination of tests for diagnosing cognitive impairment among covered members.

To address this concern, sensitivity analyses were performed and presented to allow decision-makers to better assess the budget impact on their health plans. In addition, the sensitivity of the care pathway in detecting AD was modest at 35.31% for the baseline usual care scenario and at 34.57% for the BBM scenario. These low values are due to the fact that the MMSE, which was used as the entry point into the care pathway, has less than optimal test performance, leading to a large number of patients with false negative results. Using an alternative modeling structure in which further testing for patients identified by an abnormal MMSE was used instead as the starting point for entry into the model, the sensitivity of the care pathway improved to 90.06% for the baseline usual care scenario and 90.59% for the BBM scenario. Lastly, the authors focused on MMSE testing as the entry criterion for the modeling exercise. While multiple tests are available for use, the MMSE is the most commonly administered psychometric assessment of cognitive functioning.^29^

Additionally, there are natural limitations with the cost assumptions for the deterministic baseline scenario. For this baseline scenario, the authors picked a single point estimate for each cost that the authors believed was a fair representation of the mean value.

This single point estimate therefore varies by patient subgroups such as geography, age, insurance coverage status, or provider type. To ensure that this analysis was robust to these assumptions, the authors performed the probabilistic sensitivity analysis as described above. Because the authors included patients that were aged 60 and older, not everyone in the model was Medicare eligible for coverage. The authors used this age cut-off to reflect the clinical literature, which largely focuses on the aged 60 and older population for the evaluation of cognitive decline. Although the 60–64 age group in the model would be associated with commercial insurance, which generally pays 17%–44% more than Medicare fee-for-service insurance,^41^ this younger age group also has a much lower rate of MCI and AD, suggesting that their contribution to the results of the model are modest and consistent with the range that the authors evaluated in sensitivity analysis. Additionally, 17% of patients are estimated to be dual enrollees in Medicaid and Medicare,^42^ and Medicaid reimbursement levels are estimated to be 28% lower than Medicare levels.^43^ The product of this decreased price and the proportion of the population is less than 5%, suggesting that its contribution to prices is adequately reflected by the ±10% values used in this probabilistic sensitivity analysis as described in Figure 3.

This budget impact model considered outcomes to the point of diagnosis only: further studies may address the combination of BBM testing and pharmacotherapy on patient and cost outcomes. In addition, the model presented in this study was specific to assumptions on US care delivery, as opposed to other international markets that have different care models, methods of reimbursement, technology and service costs, and priorities for disease management. The focus used here was on the budget impact analysis, which centers around affordability: this affordability theme was selected as the primary theme due to the role of budgeting on coverage and reimbursement decisions by health systems and health plans.

Additionally, the PrecivityAD blood test used in this study has not been cleared by the FDA: this test has been clinically validated and is commercially available in 49 states pursuant to Clinical Laboratory Improvement Amendments (CLIA) regulations and is used for clinical care purposes. Finally, this model was based on a decision tree that did not allow for consideration of patient wait times or resource utilization at the health care providers' office as well as assessment of negative sequelae of diagnostics, including adverse events from sample collection or distress from positive test results. Further studies are needed to address these other implementation components of BBM testing.

## Conclusions

The use of the PrecivityAD blood test before historically standard methods in patients undergoing evaluation for cognitive impairment may prevent unnecessary additional confirmatory testing, provide cost savings, and thus reduce the predicted burden on both patients and payers. BBM such as the PrecivityAD blood test can fill the gap associated with providers' shift to a more pathological diagnosis of AD for their patients with cognitive impairment, while avoiding the invasive and resource-intensive nature of usual care technology to meet the unmet needs of patients, health care providers, and health plans.
